# Chinmo affects developmental timing and patterning during cockroach embryogenesis

**DOI:** 10.1371/journal.pone.0347492

**Published:** 2026-05-08

**Authors:** Judit Gonzalvo, Jorge Escudero, Xavier Belles

**Affiliations:** Institute of Evolutionary Biology (CSIC-Universitat Pompeu Fabra), Barcelona, Spain; University of Leipzig Faculty of Life Sciences: Universitat Leipzig Fakultat fur Lebenswissenschaften, GERMANY

## Abstract

The transcription factor Chinmo (short for Chronologically Inappropriate Morphogenesis) is a key regulator of developmental timing and metamorphosis in insects, where it represses metamorphosis-related genes during postembryonic development. While the postembryonic functions of Chinmo are well established, its role during embryogenesis is poorly understood. Here, we investigate the expression pattern and function of *chinmo* during embryogenesis in a hemimetabolan insect, the German cockroach, *Blattella germanica*. qRT-PCR measurements revealed exceptionally high levels of *chinmo* transcripts at embryonic day 0 (nearly an order of magnitude higher than those observed in postembryonic stages), followed by a sharp decline throughout embryogenesis, consistent with a maternal origin of the transcripts. Maternal RNA interference targeting *chinmo* resulted in a significant reduction in hatchling numbers, and produced characteristic embryonic defects. Early embryos exhibited severe abnormalities in germ-band formation and pronounced developmental delays, particularly affecting appendage differentiation. At later-stages, embryos displayed persistent developmental arrest, defects in cuticle replacement, and abnormalities in early embryonic development. Together, these findings demonstrate that Chinmo is required for proper germ-band formation and timely developmental progression. Comparative analysis with the holometabolan species *Drosophila melanogaster* revealed marked differences in the dynamics of embryonic *chinmo* expression, which peaks during mid-to-late embryogenesis. This pattern is consistent with previous reports showing that *D. melanogaster* embryos with reduced *chinmo* expression arrest during late embryogenesis. Collectively, these data support the hypothesis that evolutionary shifts in the timing and magnitude of *chinmo* expression during embryogenesis have contributed to the divergence of developmental strategies between hemimetabolan and holometabolan insects.

## Introduction

The transcription factor Chinmo (short for Chronologically Inappropriate Morphogenesis) was first described in the fruit fly *Drosophila melanogaster* as a BTB–zinc finger protein that specifies the temporal identity of neurons during postembryonic brain development [1]. Subsequent studies demonstrated that Chinmo also plays a central role in maintaining the larval state and inhibiting holometabolan metamorphosis. This function was initially reported in *D. melanogaster* [2,3], and later extended to other holometabolan insects, including the red flour beetle *Tribolium castaneum* [4] and the fall armyworm *Spodoptera frugiperda* [5]. At the molecular level, Chinmo suppresses holometabolan metamorphosis by repressing the expression of *Broad-complex* (*BR-C*), which is required for pupal formation, and *Ecdysone-inducible protein 93F* (*E93*), which promotes adult morphogenesis [2–5]. More recently, Chinmo has been shown to repress hemimetabolan metamorphosis by inhibiting *E93* expression, but not *BR-C*, as shown in the large milkweed bug, *Oncopeltus fasciatus* [6], and the German cockroach, *Blattella germanica* [7]. The findings in hemimetabolan species suggest that repression of *BR-C* by Chinmo is a derived feature that emerged in the context of the evolution of holometaboly.

Since the nymph or larva ultimately forms during embryogenesis, investigating the functions of Chinmo in the embryo can yield valuable insights into its fundamental roles in insect development. As reported by Ylla et al. [8] and recently discussed by Truman and Riddiford [2] and Nagata and Suzuki [6], transcriptomic analyses in *B. germanica* revealed very high levels of *chinmo* expression at the onset of embryogenesis, followed by a progressive decline throughout embryo development. Notably, peak expression levels during early embryogenesis are nearly an order of magnitude higher than those detected during nymphal development, according to data from Escudero et al. [7]. Both the distinctive temporal profile of *chinmo* expression in *B. germanica* embryos and the magnitude of its early expression strongly suggest an important role for Chinmo during embryogenesis, particularly at early stages. The present study aims to elucidate the role of Chinmo during embryonic development in *B. germanica*, thereby providing new insight into the evolutionary origins and diversification of Chinmo function in insects.

## Materials and methods

### Insects and dissections

Insects used for experiments and observations were obtained from a *B. germanica* colony maintained on Panlab 125 dog chow (Rettenmaier Iberica SL Y Cia S Com) and water ad libitum. The colony was reared in darkness at 29 ± 1°C and 60–70% relative humidity. Freshly ecdysed adult females were selected and used at the ages indicated below. All experiments were conducted using mated females; mating status was confirmed by the presence of sperm in the spermatheca at the end of the observation period. For injection treatments, dissections, and tissue sampling, insects were anesthetized with carbon dioxide.

### RNA extraction and reverse transcription

Total RNA extraction was performed using RNeasy Plant minikit (QIAGEN) in the case of young oothecae (from 0- to 4-day-old) and Tissue Total RNA purification Kit (Canvax Biotech) for older oothecae (from 6- to 16-day-old). The extracted volume was lyophilized in a Fisher-Alpha 1–2 LDplus freeze-dryer. Then, it was resuspended in 8 µL of nuclease-free water, treated with DNase I (Promega), and reverse transcribed using the First Strand cDNA Synthesis Kit (Roche). RNA quantity and quality were estimated by spectrophotometric absorption at 260 nm in a Nanodrop Spectrophotometer (MicroDigital Co, Ltd).

### Quantification of mRNA levels by quantitative real-time PCR

Quantitative real-time PCR (qRT-PCR) analyses were performed using an iQ5 Real-Time PCR Detection System (Bio-Rad Laboratories) with iTaq Universal SYBR Green Supermix (Bio-Rad Laboratories). No-template controls were included in all reaction batches to monitor contamination. Primers used to amplify *chinmo* transcripts (5′–3′) were as follows: forward, CGAGCGACTTTACGGGTATG; reverse, GGCTACAAAAGGGACATGGA (sequence accession number: PV101163). Primers for the reference gene *Actin 5C* (*Act5C*) were: forward, AGCTTCCTGATGGTCAGGTGA; reverse, ACCATGTACCCTGGAATTGCCGACA (sequence accession number: AJ862721). Primer efficiency was validated for each primer pair by generating standard curves using four serial dilutions. Amplification reactions were conducted under the following thermal cycling conditions: an initial denaturation at 95°C for 3 min; 44 cycles of 95°C for 10 s and 57°C for 1 min; followed by 95°C for 10 s. Melting curve analysis was performed from 57°C to 95°C, with fluorescence measurements taken at 0.5°C increments. Transcript levels were calculated with the Bio-Rad CFX Maestro software (version 5.3.022.1030). Relative gene expression was determined using the 2^Δ^Ct method [9](see also [10]), normalized to *Act5C*, whose expression remained stable throughout the examined embryogenesis period. Results are expressed as copies of target mRNA per 1,000 copies of *Act5C* mRNA.

### Expression studies from transcriptomic libraries

Transcriptomic expression profiles of *B. germanica* were obtained from RNA-seq data published by Ylla et al. [8] and deposited in the Gene Expression Omnibus (GEO) under accession number GSE99785. This dataset comprises 22 libraries corresponding to 11 developmental stages, with two biological replicates per stage. These stages include pre-embryonic development (non-fertilized egg), five embryonic stages (0, 1, 2, 6, and 13 days after oviposition), four nymphal instars (first, third, fifth, and sixth), and the adult female. The *D. melanogaster* RNA-seq dataset used for comparative analyses was generated by the Kevin White laboratory and is available in GEO under accession number GSE18068. This dataset also comprises 22 libraries from 11 developmental stages, with two biological replicates per stage, spanning the entire embryonic period (six sequential stages: 0–4 h, 4–6 h, 6–12 h, 12–16 h, 16–20 h, and 20–24 h), the three larval instars (L1, L2, and L3), the pupal stage, and the adult female.

### RNA interference

Maternal RNA interference (RNAi) in *B. germanica* was performed as previously described [11]. Primers used to generate double-stranded RNA (dsRNA) targeting *chinmo* transcripts were (5′–3′): forward, CAGCACCACTATGTCCAAGTG; reverse, GAGTCCTGCATGGCTTCGGA, yielding a 515-nucleotide fragment (sequence accession number: PV101163). Primers used to generate the control dsRNA (dsMock), corresponding to a sequence from *Autographa californica* nucleopolyhedrosis virus, were: forward, CCTACGTGTACGACAACAAGT; reverse, ATGAAGGCTCGACGATCCTA, yielding a 441-nucleotide fragment (sequence accession number: K01149). dsRNAs were synthesized using RiboMAX Large Scale RNA Production System T7 (Promega) following the manufacturer’s instructions. Two doses of dsChinmo (12 µg each in a 1 µL volume) were injected into the abdomen of the adult female using a Hamilton 75N syringe. The first injection was administered on day 1 after the imaginal molt (AdD1), and the second on day 5 (AdD5). Control specimens were treated in parallel with equivalent doses of dsMock.

### Morphological studies and imaging

For observations on day 4 of embryogenesis, oothecae were detached from the female abdomen and incubated in PBT (0.1% Triton X-100 in 0.2 M PBS) in a water bath at 95°C. Oothecae were then artificially opened, and embryos were dechorionated and individualized. Then, they were fixed in 4% paraformaldehyde in 0.2 M PBS for 1 h, washed with PBT, and incubated in 1 mg/mL 4′,6-diamidino-2-phenylindole (DAPI) for 10 min. The embryos were finally mounted in Mowiol (Calbiochem) and examined using a Zeiss AxioImager Z1 fluorescence microscope equipped with an Apotome. For observations on day 12 of embryogenesis, oothecae were detached from the female abdomen on that day and artificially opened. The oothecae were incubated for 5 min in water at 95ºC to facilitate the individualization of the embryos. Images of the embryos were obtained using a Zeiss DiscoveryV8 stereomicroscope (Carl Zeiss MicroImaging.

### Statistics

Quantitative data are presented as the mean ± standard error of the mean (SEM). Statistical analyses of qRT-PCR data were performed using GraphPad Prism version 8.1.0 for Windows (GraphPad Software). Data were evaluated for homogeneity and normality of variance using the Shapiro–Wilk test; all datasets met the assumptions of normality and did not require transformation. Statistical significance between control and treated groups was determined using Student’s *t*-test.

## Results

### *chinmo* transcript levels are markedly elevated during early embryogenesis

**Fig 1:**
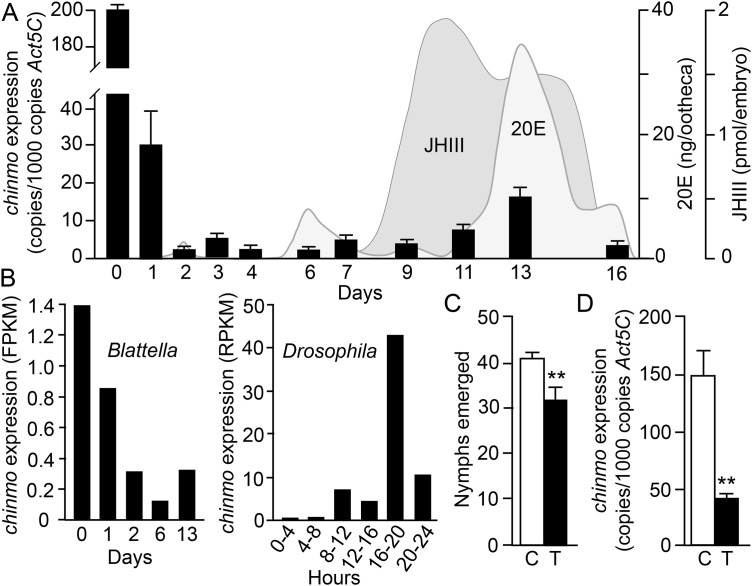
Expression of *chinmo* in embryos of *Blattella germanica* and effects of maternal RNAi on hatchling numbers. (A) transcript levels of *chinmo* in embryos at days 0, 1, 2, 3, 4, 6, 7, 9, 11, 13, and 16, measured by qRT-PCR; superimposed are the temporal profiles of 20-hydroxyecdysone (20E) and juvenile hormone III (JH III), according to Maestro et al. [16] and Maestro et al. [17], respectively. **(B)** Transcript levels of *chinmo* during embryogenesis of *Blattella germanica* and *Drosophila melanogaster*, obtained from previously reported whole-body transcriptomes (see [8]). **(C)** Number of nymphs emerging from oothecae of females treated with dsChinmo and from control females. **(D)** Expression of *chinmo* in day 0 embryos from adult females treated with dsChinmo and from controls. In panels **(A)** and **(D)**, each measurement represents three **(A)** or four **(D)** biological replicates, and results are expressed as copies of *chinmo* per 1,000 copies of *Act5C* mRNA; data are presented as mean ± SEM. In panel **(C)**, the results represent 16 oothecae for controls and 21 oothecae for dsChinmo-treated females. In panels **(C)** and **(D)**, two asterisks indicate statistically significant differences compared with controls (Student’s t-test, p < 0.01). The individual values corresponding to the data shown in panels **A**, **C**, and **D** are provided in Tables S1, S2, and S3, respectively, in the Supporting Information.

To characterize *chinmo* expression during *B. germanica* embryogenesis, transcript levels were quantified in oothecae by qRT-PCR at days 0, 1, 2, 3, 4, 6, 7, 9, 11, 13, and 16. As shown in Fig 1A (see also Table S1), highest levels of *chinmo* transcripts were measured at day 0, reaching approximately 200 copies of *chinmo* mRNA per 1,000 copies of *Act5C* mRNA. Transcript levels decreased by nearly one order of magnitude by day 1, and remained low throughout the remainder of embryogenesis, with values exceeding 10 copies per 1,000 *Act5C* mRNA only at days 11 and 13.

This expression profile matches that previously obtained from transcriptomic analyses [8] (Fig 1B). Notably, *chinmo* transcript levels in day-0 embryos are almost an order of magnitude higher than the maximal levels detected during nymphal development, which peak at approximately 40 copies per 1,000 A*ct5C* mRNA copies in the wing pads of fourth-instar nymphs [7]. Together, these data indicate that *chinmo* is highly expressed during early embryogenesis and suggest a functional role for Chinmo at this developmental stage.

### Maternal RNAi of Chinmo reduces the number of hatchlings

To investigate the role of Chinmo during early embryogenesis, maternal RNAi was performed. Adult females of *B. germanica* were injected with two doses of 12 µg of dsRNA targeting *chinmo* transcripts (dsChinmo), administered on day 1 after the imaginal molt (AdD1) and again on day 5 (AdD5). Females were then allowed to mate; successful fertilization was confirmed at the end of the experiment by the presence of spermatozoa in the spermatheca. Control females were treated in parallel with a non-specific dsRNA (dsMock). Both experimental groups were monitored through ootheca formation and until the emergence of first-instar nymphs. In both treatments, hatching occurred 19 days after ootheca formation. Othecae from control females produced an average of 41.06 ± 0.77 nymphs (n = 16 oothecae), whereas those from dsChinmo-treated females produced an average of 32.19 ± 2.17 nymphs (n = 21 oothecae), which represents a mean reduction of 22% (Fig 1C, Table S2). To assess the efficiency of maternal RNAi, *chinmo* mRNA levels were measured in day-0 embryos, corresponding to the peak of *chinmo* expression. In embryos from dsChinmo-treated females, *chinmo* transcript levels were reduced by 71% relative to controls (Fig 1D, Table S3), demonstrating that maternal RNAi efficiently depleted *chinmo* transcripts.

### Depletion of Chinmo impairs embryo development

The reduced number of hatchlings observed in oothecae from dsChinmo-treated females compared with controls suggests that Chinmo depletion disrupts embryonic development. To identify the embryogenic processes most affected, maternal RNAi was performed as described above, with dsRNA injections on AdD1 and AdD5. Then, embryos were dissected and analyzed at two key developmental stages: day 4 and day 12 of embryogenesis. At day 4, the rudiments of the cephalic and thoracic regions are differentiated, abdominal segmentation begins, and the tail starts folding [8,12], corresponding to stage 6 of Tanaka [13]. At day 12, embryos closely resemble first-instar nymphs, with completed dorsal closure, onset of eye pigmentation, and distal portions of the antennae and legs extending to the fourth abdominal segment [8,12], corresponding to stage 14 of Tanaka [13].

**Fig 2:**
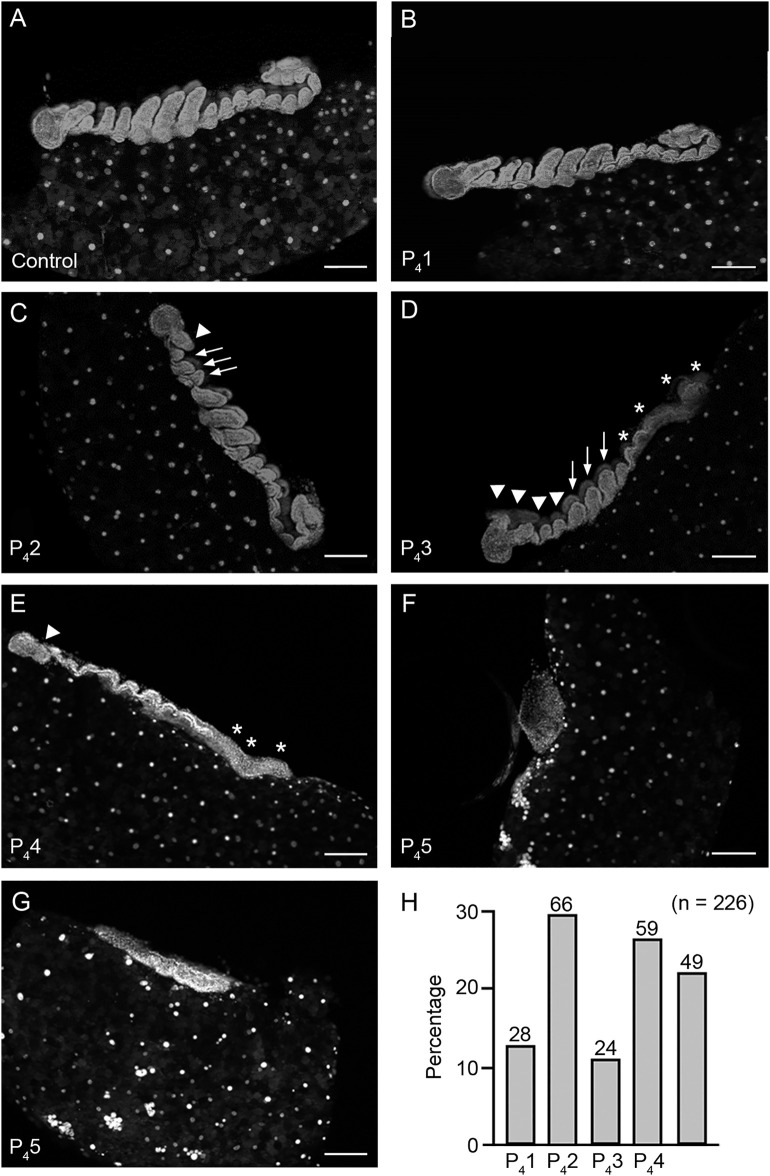
Phenotypic effects of maternal RNAi of *chinmo* in *Blattella germanica* at day 4 of embryogenesis. **(A)** Morphology of a control embryo at day 4. **(B)** Phenotype P_4_1, embryos indistinguishable from 4-day-old controls. **(C)** Phenotype P_4_2, embryos with normal overall development but with antennae (arrowhead) and mouthparts (arrows) shorter than those of controls. **(D)** Phenotype P_4_3, embryos with a poorly differentiated cephalic region (arrowheads), underdeveloped thoracic appendages (arrows), and an unsegmented abdomen (asterisks). **(E)** Phenotype P_4_4, embryos with rudimentary overall segmentation, particularly in the cephalic (arrowhead) and abdominal (asterisks) regions. **(F–G)** Phenotype P_4_5, embryos with severe defects in germ band formation, appearing as a condensed amorphous mass **(F)** or short, unsegmented structures **(G)**. **(H)** Percentage of embryos exhibiting phenotypes P_4_1–P_4_5; the total number of embryos analyzed was 226, and the number of embryos observed in each category is indicated above each bar. In panels A–E, the left side of each image corresponds to the cephalic region of the embryo. Scale bars in panels **A**-**G** represent 200 µm.

Day-4 embryos were analyzed from 11 oothecae laid by dsChinmo-treated females, with 10–42 embryos examined per ootheca (226 embryos in total). As controls, 8 oothecae from dsMock-treated females were analyzed, examining 22–35 embryos per ootheca (187 embryos in total). Embryos from control oothecae displayed normal morphology for this developmental stage, corresponding to stage 6 of Tanaka [13] (Fig 2A). In contrast, embryos from dsChinmo-treated females exhibited a spectrum of phenotypes that were classified into five categories (P_4_1–P_4_5). P_4_1 embryos (Fig 2B) were morphologically indistinguishable from 4-day-old control embryos. P_4_2 embryos showed overall normal patterning, but the cephalic appendages, antennae (Fig. 2C, arrowhed) and mouthparts (Fig 2C, arrows) were shorter than in controls, consistent with a slight developmental delay. P_4_3 embryos exhibited a poorly differentiated cephalic region (Fig 2D, arrowheads), underdeveloped thoracic appendages (Fig 2D, arrows), and an unsegmented abdomen (Fig 2D, asterisks), corresponding to an intermediate stage between 3 and 4 days of development. P_4_4 embryos displayed rudimentary overall segmentation, particularly in the cephalic (Fig 2E, arrowhead) and abdominal (Fig 2E, asterisks) regions. Finally, P_4_5 embryos showed severe defects in germ-band formation, appearing either as a condensed amorphous mass (Fig 2F) or as a short, elongated but unsegmented structure (Fig 2G). Quantitatively, unaffected embryos (P_4_1) represented 12.4% of the total. Embryos in categories P_4_2, P_4_3, and P_4_4, which displayed largely normal patterning but varying degrees of developmental delay, accounted collectively for 65.9%. Severely affected embryos (P_4_5), exhibiting profound germ-band defects and likely unviability, represented the remaining 21.7% (Fig 2H).

**Fig 3:**
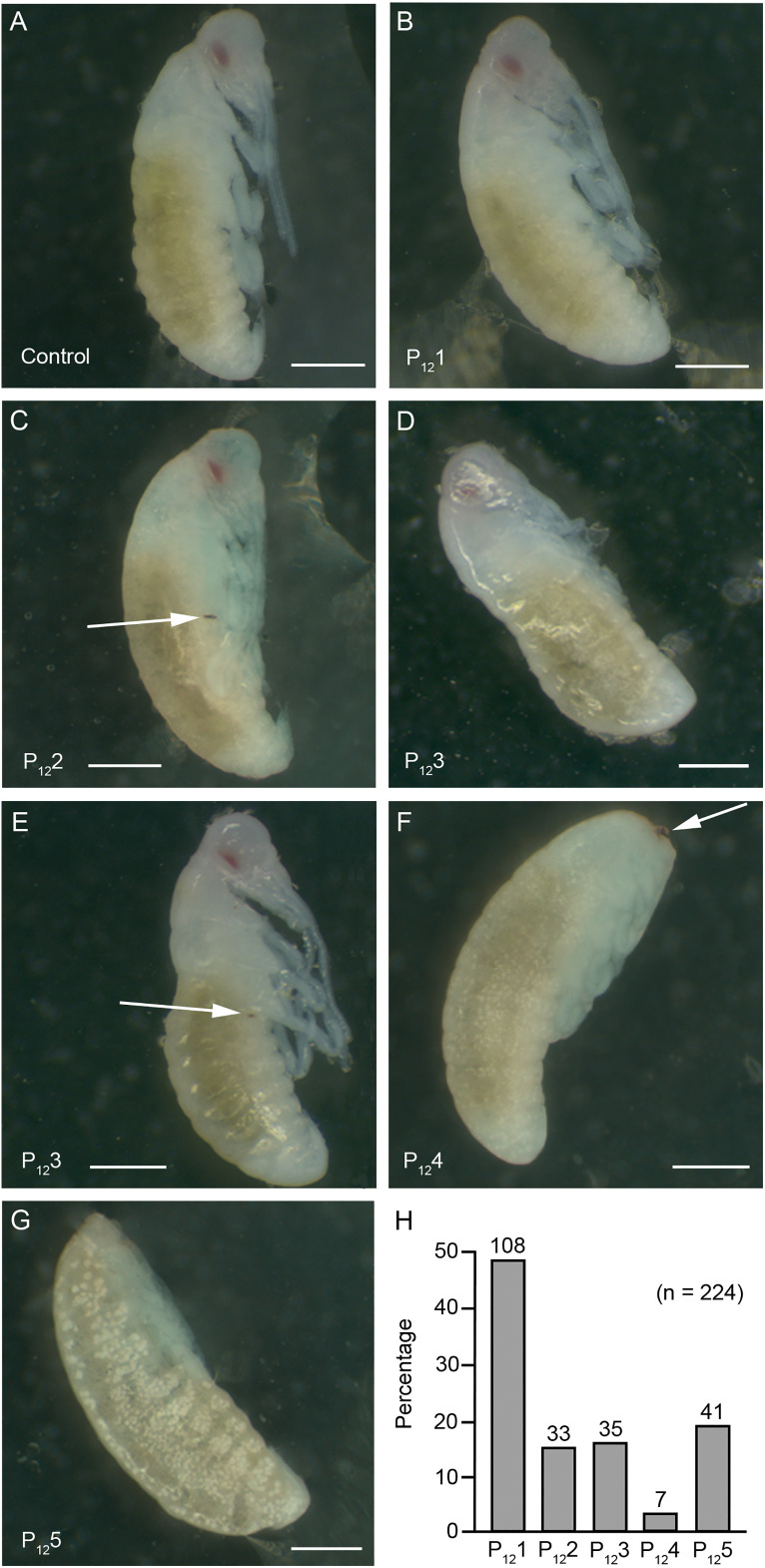
Phenotypic effects of maternal RNAi of *chinmo* in *Blattella germanica* at day 12 of embryogenesis. **(A)** Morphology of a control embryo at day 12. **(B)** Phenotype P_12_1, embryos indistinguishable from 12-day-old controls. **(C)** Phenotype P_12_2, embryos with normal overall development but with pleuropodia present and sclerotized (arrow), and appendages somewhat shorter than those of controls. **(D–E)** Phenotype P_12_3, embryos morphologically normal completely **(D)** or partially **(E)** enclosed within the second embryonic cuticle. **(F)** P_12_4 Embryos with poorly developed appendages, and with the eyes less pigmented and improperly positioned. **(G)** P_12_5 embryos consisting of an almost amorphous mass lacking clear segmentation, at most exhibiting faint indications of abdominal segments. **(H)** Percentage of embryos exhibiting phenotypes P_12_1–P_12_5; the total number of embryos analyzed was 224, and the number of embryos observed in each category is indicated above each bar. Scale bars in panels **A**-**G** represent 500 µm.

Day-12 embryos were examined from 11 oothecae laid by dsChinmo-treated females, with 7–43 embryos analyzed per ootheca (224 embryos in total). As controls, 8 oothecae from dsMock-treated females were analyzed, examining 20–32 embryos per ootheca (173 embryos in total). Control embryos exhibited normal morphology for this stage, corresponding to stage 14 of Tanaka [13] (Fig 3A). Among embryos from dsChinmo-treated females, five phenotypic categories (P_12_1–P_12_5) were identified. P_12_1 embryos (Fig. 3B) were morphologically indistinguishable from control embryos. P_12_2 embryos exhibited overall normal morphology but retained sclerotized pleuropodia (Fig 3C, arrow) and had slightly shortened appendages compared with controls (Fig 3C). P_12_3 embryos appeared morphologically normal but were completely (Fig 3D) or partially (Fig 3E) enclosed within the second embryonic cuticle; some embryos retained the pleuropodia (Fig 3E, arrow). P_12_4 embryos displayed poorly developed appendages, with eyes that were weakly pigmented and incorrectly positioned (Fig 3F). P_12_5 embryos consisted of a yolk mass lacking appendage differentiation and clear segmentation; at most they presented blurred incipient appendages and faint indications of abdominal segmentation (Fig 3G). Quantitative analysis revealed that 48.2% of embryos belonged to category P_12_1 and were indistinguishable from controls. P_12_2 embryos free form the second embryonic cuticle but retaining pleuropodia represented 15.6%, while P_12_3 embryos, enclosed within the second embryonic cuticle, and in some cases retaining the pleuropodia accounted for 14.8%. Embryos exhibiting severe developmental defects (P_12_4 and P_12_5) together accounted for 21.4% of the total (Fig 3H).

## Discussion

### *chinmo* transcripts at embryonic day 0 are maternal and far exceed postembryonic levels

Analysis of *chinmo* expression during *B. germanica* embryogenesis by qRT-PCR revealed exceptionally high transcript levels at day 0. Transcript abundance then declined by nearly one order of magnitude and remained low, with only minor fluctuations, throughout the remainder of embryonic development (Fig 1A). This expression profile is similar to that obtained from embryonic transcriptomic datasets of *B. germanica* reported by Ylla et al. [8], which also show a pronounced peak of *chinmo* expression at day 0 (Fig 1B). Elevated *chinmo* transcript levels in *B. germanica* embryos were reported by Chafino et al. [3]; however, the developmental stage of the embryos analyzed in that study was not specified. When comparing our expression profile (Fig 1A) with expression data in postembryonic stages [7], *chinmo* expression at embryonic day 0 is markedly higher than that detected in nymphal tissues. Even in wing pads, the postembryonic tissue exhibiting the highest *chinmo* expression [7], transcript levels are approximately one order of magnitude lower than those observed at embryonic day 0. Collectively, these observations indicate that *chinmo* is most abundantly expressed during the earliest stages of embryogenesis, suggesting a prominent role for Chinmo at this developmental window.

In *B. germanica*, the maternal-to-zygotic transition occurs between embryonic days 0 and 2 [8,14]. The timing of this transition strongly suggests that the high levels of *chinmo* transcripts detected at day 0 are of maternal origin. Moreover, comparison of *chinmo* expression dynamics with the embryonic concentration profiles of the two principal insect developmental hormones, JH and 20E, reveals a temporal coincidence between an increase of *chinmo* expression on day 13 and a peak of 20E, in a context of high JH levels. In *S. frugiperda* Sf9 cells, treatment with the JH analog Methoprene did not affect *chinmo* expression, whereas 20E increased it by approximately 1.4-fold. Combined treatment with Methoprene and 20E produced a 2.6-fold increase in *chinmo* expression [5], suggesting that 20E enhances *chinmo* activity in the presence of JH. A similar interaction may also occur on day 13 of *B. germanica* embryogenesis.

In *D. melanogaster*, immunocytochemical analyses have shown that Chinmo becomes detectable at approximately embryonic stage 14, coinciding with germ-band retraction and dorsal closure. The protein is initially restricted to the brain and ventral nerve cord and subsequently expands to all tissues, becoming ubiquitous by hatching [2]. The onset of Chinmo detection in *D. melanogaster* at stage 14 (approximately 11 h after fertilization) is consistent with staged transcriptomic data showing a significant increase in *chinmo* expression between 8 and 12 h after fertilization [8] (Fig 1B).

### Embryonic Chinmo functions are primarily associated with germ band formation and developmental timing

Analysis of embryos from *B. germanica* females subjected to maternal *chinmo* RNAi revealed two major phenotypic outcomes at day 4 of development. The first involved severe defects in germ-band formation, which appeared markedly reduced or malformed (phenotype P_4_5), affecting 22% of embryos. The second consisted of a generalized developmental delay, most evident in the formation of appendages, particularly the cephalic appendages (P_4_2 + P_4_3 + P_4_4, 66%) (Fig 2). The impairment of germ-band formation is consistent with the pronounced peak of *chinmo* expression observed at embryonic day 0 in *B. germanica*. In contrast, *chinmo* expression in *D. melanogaster* reaches its highest levels during the final quarter of embryogenesis, approximately 75% of development (Fig 1B). Nevertheless, *chinmo* mutants in *D. melanogaster* are embryonic lethal: when the *chinmo*¹ mutant balanced over the CyO line (*chinmo*¹/CyO) was selfcrossed, approximately one-quarter of the eggs showed no signs of embryogenesis, likely corresponding to CyO/CyO embryos [2]. Interestingly, another quarter of eggs arrested during late embryogenesis [2]. These late-arresting embryos had reduced *chinmo* expression and exhibited two characteristic phenotypes: fully developed embryos with gas-filled tracheae, and embryos displaying sclerotized mouth hooks and clearly defined denticle belts, but having gut structures corresponding to earlier developmental stages [2]. The generalized developmental delay observed in Chinmo-depleted *B. germanica* embryos is noteworthy in light of the established role of Chinmo as a regulator of developmental timing during postembryonic stages [1–5,7]. In postembryonic contexts, Chinmo depletion or suppression typically accelerates developmental transitions, whereas during *B. germanica* embryogenesis its depletion leads, among other effects, to developmental delay. However, precedents for this apparent discrepancy exist. As we mentioned above, in *D. melanogaster*, embryos arrested late in development with low levels of Chinmo immunoreactivity, despite displaying sclerotized mouth hooks and well-defined denticle belts, exhibited delayed gut configuration [2], suggesting that Chinmo may exert context-dependent effects on developmental timing.

From day-4 observations of embryos from females treated with dsChinmo, two major classes of developmental impairment were identified. First, severe defects in germ-band formation (P_4_5, 21.7%), and second, varying degrees of developmental delay (P_4_2 + P_4_4, 65.9%) (Fig 2). By day 12 of embryogenesis, three distinct phenotypic classes were apparent. One class showed severe developmental abnormalities (P_12_4 + P_12_5, 21.4%); a second class appeared morphologically normal but remained enclosed within the second embryonic cuticle (P_12_3, 16%); and a third class exhibited developmental delay accompanied by retention of the pleuropodia (P_12_2, 15.6%) (Fig 3). The proportion of severely affected embryos at day 4 (P_4_5, 21.7%) closely matches the frequency of severely defective embryos at day 12 (P_12_4 + P_12_5, 21.4%), and corresponds well with the 22% reduction in hatchlings (Fig 1C), indicating that impaired germ-band formation is the primary cause of embryonic lethality. Most embryos displaying the P_12_2 and P_12_4 phenotypes are likely viable, as they may recover from early developmental delays. The P_12_3 phenotype is associated to the elimination of the second embryonic cuticle, which forms around day 6 of development and coincides with the second peak of 20E (Fig 1A). P_12_3 embryos may ultimately complete development if this cuticle is removed during the third 20E peak, occurring around day 13 (Fig 1A), which triggers formation of the nymphal cuticle. Collectively, these findings indicate that embryonic Chinmo functions are tightly associated with germ-band formation and the regulation of developmental timing in *B. germanica*.

### The expression pattern of *chinmo* in cockroaches and flies suggests distinct embryonic functions in hemimetabolan and holometabolan insects

The distinct temporal patterns of *chinmo* expression during embryogenesis in *B. germanica* and *D. melanogaster* suggest that Chinmo may fulfill different embryonic functions in hemimetabolan and holometabolan insects. Consistent with this notion, phenotypic analyses of embryos with reduced *chinmo* expression reveal marked differences between *B. germanica* (this study) and *D. melanogaster* [2]. In *B. germanica*, our observations indicate that Chinmo plays a role in germ-band formation and influences early developmental timing, consistent with its high expression on day 0 (Fig 1A). It also has a later function in the elimination of the second embryonic cuticle, which occurs after day 6. In contrast, most *D. melanogaster* embryos with reduced *chinmo* expression arrest during late development [2], in agreement with the high expression levels observed during the final third of embryogenesis (Fig 1B).

**Fig 4:**
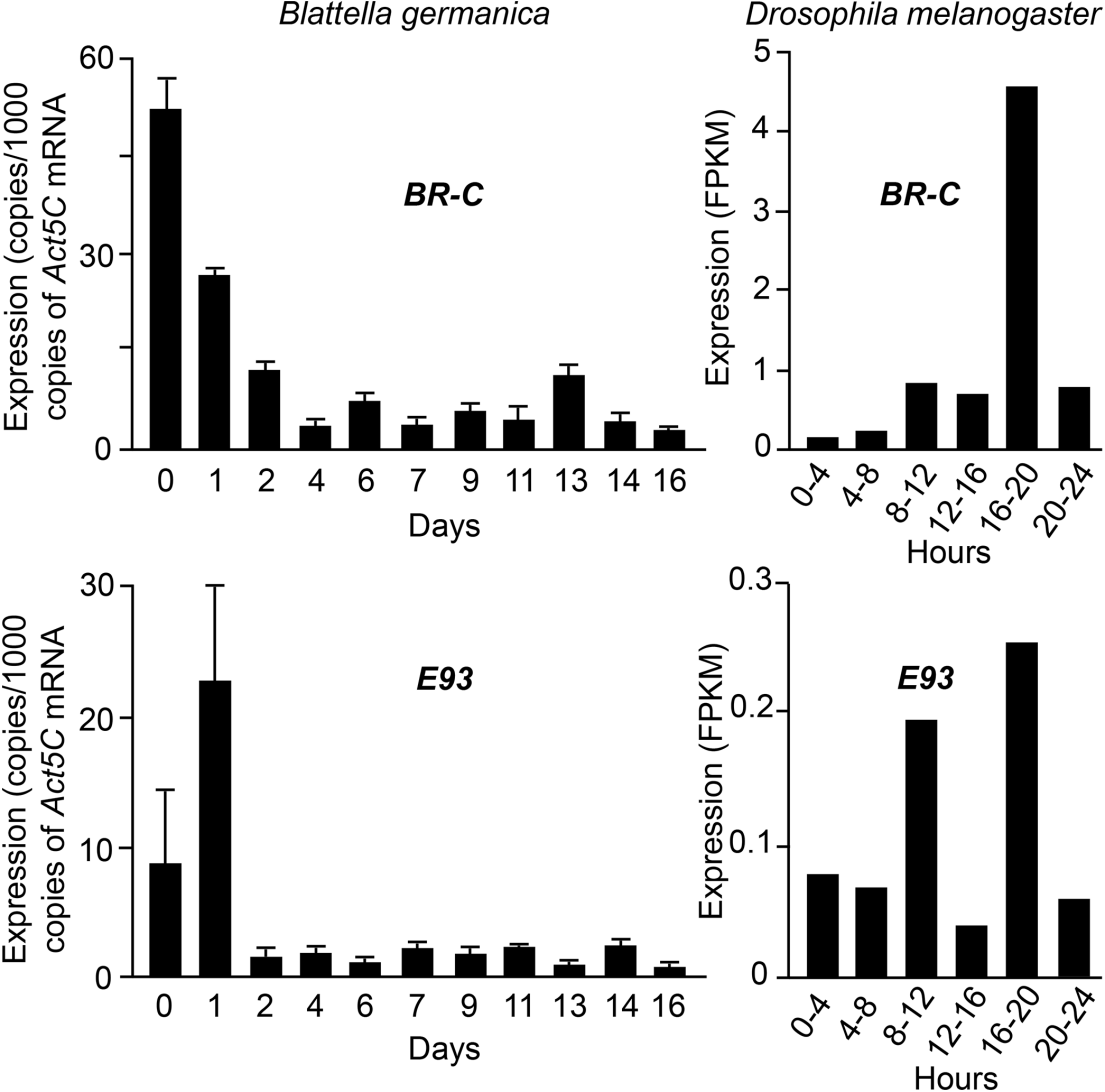
Expression levels of *Broad-complex* (*BR-C*) and *Ecdysone-inducible protein 93F* (*E93*), during embryogenesis of *Blattella germanica* and *Drosophila melanogaster*. qRT-PCR data of *B. germanica* are from Fernandez-Nicolas et al. [18] (*BR-C*) and Fernandez-Nicolas et al. [15] (*E93*). Transcriptomic data of *D. melanogaster* are from White laboratory, available in GEO under accession number GSE18068 (see [8]).

The expression pattern of *chinmo* in *B. germanica*, characterized by a pronounced peak at embryonic day 0 (ED0), is reminiscent of that observed for *BR-C*, which also shows a marked maximum at ED0 [15]. It is also partially similar to that of *E93*, whose highest expression occurs at ED1, following lower levels at ED0, suggesting a transition from maternal to zygotic transcription [16] (Fig 4). In contrast, *BR-C* display maximal expression during mid-to-late embryogenesis in *D. melanogaster*, while the expression of *E93* in this species is extremely low, so that the apparent fluctuations measured (Fig 4) do not appear to be significant. We previously reported that the embryonic expression patterns of *E93* in *B. germanica* (a large peak in early embryo) and *D. melanogaster* (low values in the same period) are broadly representative of hemimetabolan and holometabolan species, respectively, leading us to propose that the reduction of *E93* expression in early embryos may have contributed to the evolution of holometaboly [15]. Whether the embryonic expression patterns of *BR-C* in these two species are similarly generalizable across hemimetabolan and holometabolan lineages remains to be determined. Taken together, the available data support the hypothesis that evolutionary shifts in the timing and magnitude of *chinmo* expression during embryogenesis have contributed to the divergence of developmental strategies between hemimetabolan and holometabolan insects.
